# Fr-AGILE: validation and reliability of a multidimensional and rapid frailty assessment tool in the Turkish elderly population

**DOI:** 10.1007/s40520-025-03200-7

**Published:** 2025-10-30

**Authors:** Ege Ileri Istanbullu, Hande Selvi Oztorun, Ertugrul Demirel, Rana Tuna Dogrul, Gunes Arik, Sevil Uygun Ilikhan, Kamile Silay, Selma Karaahmetoglu

**Affiliations:** 1 Department of Internal Medicine, Ankara Bilkent City Hospital, Ankara, Türkiye Turkey; 2 Department of Geriatrics, Ankara Bilkent City Hospital, Ankara, Türkiye Turkey; 3 Department of Geriatrics, Ankara Yildirim Beyazit University, Ankara, Türkiye Turkey

**Keywords:** Fr-AGILE, Frailty, Geriatrics, Comprehensive geriatric assessment, Older adults, Sarcopenia, Validation study

## Abstract

**Purpose:**

With the global aging population, the prevalence of frailty is increasing, making its timely and accurate identification critical for prevention. This study aimed to evaluate the reliability and validity of the Fr-AGILE scale—a rapid, multidimensional frailty assessment tool—for use in the Turkish geriatric population.

**Methods:**

A total of 101 individuals aged 65 and over who visited a geriatric outpatient clinic were enrolled. All participants were assessed using the Fr-AGILE scale, alongside the FRAIL and Clinical Frailty Score as reference standards.

**Results:**

The Fr-AGILE scale demonstrated a sensitivity of 71.4% and specificity of 94.1%. Its positive predictive value was 83.3%, and the negative predictive value was 56.1%. High inter-rater reliability was observed, with substantial agreement in the physical, mental, and nutritional domains, and excellent agreement in the socioeconomic domain. Internal consistency coefficients were strong across all domains. The Fr-AGILE score showed significant correlations with the FRAIL scale (*r* = 0.742), Clinical Frailty Score(*r* = 0.732), and Comprehensive Geriatric Assessment with an AUC of 0.906 for severe frailty (*p* < 0.001).

**Conclusion:**

The Fr-AGILE scale is a valid and reliable tool for rapid, multidimensional frailty assessment in older adults. It provides a practical and efficient method for identifying frailty across physical, mental, nutritional, and socioeconomic domains in Turkish geriatric patients.

## Introduction

With the global aging of the population, the concept of frailty has emerged as a critical marker of functional status in older adults. Although there is no universally accepted definition, frailty is generally regarded as a clinical syndrome marked by increased vulnerability to adverse health outcomes due to age-related decline across four key domains: physical, mental, nutritional, and socio-economic [1].

Frailty is considered a dynamic and potentially reversible condition. Therefore, its early identification and appropriate screening are crucial to prevent progression. As frailty increases, the risk of hospitalization, falls, disability, and long-term care needs also rises [2]. Unlike other clinical conditions, adverse outcomes such as morbidity and mortality can be more readily monitored in frail individuals.

In clinical practice, tailoring healthcare services based on the degree of frailty has been shown to improve outcomes across all levels of prevention—primary, secondary, and tertiary. In a Canadian survey involving 365 healthcare professionals, two-thirds reported that frailty assessment was helpful in identifying vulnerability, managing daily living activities, and addressing disability among the elderly [3]. Numerous studies confirm that timely and appropriate frailty assessment, especially in patients with comorbidities, is associated with reduced mortality and improved clinical outcomes [4].

The prevalence of frailty is rising rapidly, making its timely, accurate, and efficient detection a growing clinical challenge. Several validated tools are available for frailty assessment, including the Comprehensive Geriatric Assessment (CGA), the Fried frailty phenotype, the FRAIL scale, and the Clinical Frailty Scale developed by Rockwood [5–7]. While these tools have proven clinical utility, their application—particularly in outpatient settings—is often limited by time and resource constraints. Moreover, tools that are quick to administer frequently lack a comprehensive approach, as they may fail to assess all critical domains of frailty, such as physical, mental, nutritional, and socio-economic aspects.

The Frailty Index (FI) is a cumulative measure that captures age-related health deficits, including symptoms, comorbidities, laboratory parameters, and functional limitations. Multiple studies have demonstrated that the FI predicts adverse clinical outcomes more accurately than many other tools, both in hospitalized and community-dwelling older adults. To enhance its practicality in clinical settings, a modified version known as the Italian Frailty Index (IFi) was developed and validated [8]. This version expanded upon earlier models by incorporating additional items targeting the mental, nutritional, and socio-economic domains, which were previously underrepresented. Despite its comprehensiveness and proven reliability, a Major limitation of the IFi is its length; it includes approximately 40 items and is time-consuming to administer in routine clinical practice.

In 2020, the *fr-AGILE* scale was developed by selecting the most efficient and comprehensive components from the Italian Frailty Index (IFi), addressing the need for a rapid and multidimensional frailty assessment tool [9]. Validity and reliability studies conducted in the Italian population, including COVID-19 patients, demonstrated that *fr-AGILE* is an effective instrument for screening multidimensional frailty and can predict mortality, disability, and hospitalization [8, 10]. However, a validated Turkish version of this practical and reliable scale has not yet been available.

Therefore, the aim of this study was to evaluate the reliability and validity of the *fr-AGILE* scale in the Turkish geriatric population. This tool, designed to provide rapid and multidimensional assessment of frailty, is particularly important given the impact of frailty on outcomes such as hospitalization, disability, and mortality in older adults.

## Materials and methods

### Participants

This study included 101 participants who met the eligibility criteria and were evaluated at the Geriatrics Outpatient Clinic of Ankara Bilkent City Hospital. Inclusion criteria were as follows: being aged 65 years or older, the cognitive and physical ability to understand and complete the *fr-AGILE* scale and other assessment tools, and voluntary participation.

Exclusion criteria were: Not meeting the inclusion criteria, Presence of active malignancy, Physical disabilities (e.g., limb amputation, stroke sequelae, speech or hearing impairment), Acute infections, Acute illnesses (e.g., decompensated heart failure, recent myocardial infarction, cerebrovascular event, or COPD exacerbation), Hospitalization or surgical intervention within the past month, Advanced or terminal-stage disease, Inability to provide a medical history or cooperate during evaluation, Diagnosed organic psychiatric or degenerative neurological disorders.

Sociodemographic and clinical data were collected from all participants, including educational status, smoking and alcohol use, gender, height, weight, living arrangement, number of falls and hospitalizations in the past year, presence and type of urinary incontinence, vaccination status, number of medications used, and existing comorbidities.

Walking speed and hand grip strength were assessed during the physical examination. Grip strength was measured using a hand dynamometer (Takei A5401, Japan). Participants were first asked which hand they used for daily activities requiring strength (e.g., writing, eating) to identify the dominant hand. Measurements were taken from the dominant hand while the participant stood upright with the elbow and wrist fully extended. Grip strength was measured three times at five-second intervals, and the average value (in kilograms) was recorded.

For the walking speed test, participants were instructed to walk 4.57 m at their usual pace. The test was repeated twice, and the fastest time (in seconds) was used. Walking speed was calculated in meters per second by dividing the distance by the recorded time.

### Fr-AGILE scale

According to Park et al. [11], the Fr-AGILE scale is a multidimensional frailty assessment tool designed for individuals aged 65 years and older. It evaluates four core domains of frailty: physical, mental, nutritional, and socio-economic. The scale consists of 10 items distributed across these domains.

For the **physical domain**, the following questions are included: “Do you feel that you have to exert strength for everything?” and “Do you need help going up and down the stairs?” Each affirmative answer is scored as 1 point; negative answers receive 0 points. In addition, **hand grip strength** is evaluated. Based on Turkish cut-off values, grip strength ≤ 22 kg for women and ≤ 32 kg for men results in a score of 0; otherwise, 1 point is awarded [12].

The **mental domain** includes two items: **time disorientation** (participants are asked to state the current day, month, and year) and **delayed recall** (participants are asked to recall the words “bread-house-cat” presented earlier in the questionnaire). A correct response to each item scores 1 point.

For the **nutritional domain**, participants are asked: “Have you experienced unintentional weight loss of 4.5 kg or more in the last year?” and “Do you need assistance while eating?” Each affirmative response is given 1 point.

The **socio-economic domain** consists of two items: “Do you receive financial assistance from family members?” and “Do you receive physical assistance from family members?” Again, each “yes” response scores 1 point.

The total score ranges from 0 to 10 and is interpreted as follows:

- **0–3 points**: Mild frailty.
- **4–7 points**: Moderate frailty.
- **8–10 points**: Severe frailty.

Permission for the Turkish validity and reliability study of the scale was obtained from the corresponding author of the original version [8].

### Reference Tools

As part of the comprehensive geriatric assessment (CGA), all participants underwent a standardized evaluation, including medical history, physical examination, geriatric syndrome screening, and functional performance tests. The following validated tools were used:

- Katz Index of Activities of Daily Living (ADL) [13, 14].
- Lawton–Brody Instrumental Activities of Daily Living (IADL) [15].
- Mini-Mental State Examination (MMSE) [16, 17].
- Mini Nutritional Assessment (MNA) [18, 19].
- Yesavage Geriatric Depression Scale (GDS) [20].
- 4.57-meter gait speed test.

These tests are routinely administered by a trained geriatrician in the outpatient clinic.

In addition to these tools, the **FRAIL Scale** and **Clinical Frailty Scale (CFS)** were used as reference measures to assess frailty:

- The **FRAIL Scale** consists of five domains: fatigue, resistance, ambulation, illnesses, and weight loss. Scores range from 0 to 5:
  - 0 = robust.
  - 1–2 = pre-frail.
  - ≥ 3 = frail This scale has been validated for use in the Turkish population [21].
- The **Clinical Frailty Scale** categorizes patients into nine levels of frailty, ranging from 1 (very fit) to 9 (terminally ill), based on overall health status, functional capacity, and comorbidities [7]. It has also been validated in Turkish clinical settings [22].

### Translation

The original 10-item Fr-AGILE scale was first translated from English into Turkish by two academicians specializing in geriatrics, who are fluent in English and native Turkish speakers. To ensure linguistic accuracy and cultural appropriateness, a panel of experts reviewed the initial translation. Following this, the Turkish version was back-translated into English by a translator familiar with the original scale. Discrepancies were discussed among the expert panel and translators to finalize the Turkish version of the Fr-AGILE scale. The translation process followed the standardized forward–backward translation methodology [23].

Inter- and intra-observer agreement was evaluated. To assess inter-rater reliability, the Fr-AGILE scale was administered on the same day to a group of patients by a second, blinded researcher. The required sample size for this evaluation was determined by power analysis. For test–retest reliability, the same patients completed the scale again 7 to 15 days later under similar outpatient clinic conditions. The FRAIL scale and the Clinical Frailty Scale were administered separately by another experienced geriatrician. The final Turkish version of the scale is included in the supplementary material.

### Ethics

Ethical approval for this study was obtained from the Ankara City Hospital Clinical Research Ethics Committee No. 1 (Decision No: E1-23-3872, dated 16 August 2023).

### Statistical analysis

All statistical analyses were performed using IBM SPSS Statistics for Windows, Version 26.0 (IBM Corp., Armonk, NY, USA). The normality of data distribution was assessed using both visual (histograms and probability plots) and analytical methods (Kolmogorov-Smirnov and Shapiro-Wilk tests).

Descriptive statistics were reported as means and standard deviations for normally distributed variables, and as medians with minimum–maximum ranges for non-normally distributed variables. Categorical variables were expressed as frequencies and percentages.

Inter-rater reliability of the Fr-AGILE scale was assessed in 25 participants using the **Kappa coefficient**, where values ≥ 0.80 were interpreted as indicating perfect agreement. Internal consistency was evaluated using **Cronbach’s alpha**. For the FRAIL scale, the **Kuder-Richardson Formula 20 (KR-20)** and item-total correlation analyses were used; values above 0.70 were considered acceptable.

Correlations between the Fr-AGILE, FRAIL scale, Clinical Frailty Score, MMSE, and other numerical variables were analyzed using the **Spearman correlation coefficient**. Test-retest and inter-rater reliability were further evaluated using the **Intraclass Correlation Coefficient (ICC)**.

Comparisons of quantitative variables (e.g., gender, education) across Fr-AGILE groups (normal, mild, moderate, severe frailty) were conducted using the **Chi-square test** or **Fisher’s exact test** for categorical data. For continuous variables, **one-way ANOVA** was used when normal distribution was confirmed, while the **Kruskal-Wallis test** was used otherwise. Pairwise comparisons were performed using the **Student’s t-test** (after ANOVA) or **Mann–Whitney U test** (after Kruskal–Wallis), with **Bonferroni correction** applied for multiple testing. Given the three frailty groups (mild, moderate, severe), four pairwise comparisons were made, and the adjusted significance level was set at *p* < 0.0125.

To evaluate the diagnostic accuracy of the Fr-AGILE scale, **receiver operating characteristic (ROC)** curve analysis was performed, classifying patients as severely frail or non-severely frail. An **Area Under the Curve (AUC)** between 0.70 and 0.90 was considered acceptable, while AUC values close to 1 indicated excellent diagnostic performance. A **Type I error level of 5% (p< 0.05)** was used to determine statistical significance.

## Results

A total of 111 patients who presented to the Geriatrics Outpatient Clinic were initially evaluated. Nine patients were excluded due to inability to perform the walking test, and one patient was excluded due to a diagnosis of Malignancy. Thus, the final study population consisted of 101 participants.

The mean age of the included patients was 77.12 ± 6.39 years, and 61.4% (*n* = 62) were female. The mean Fr-AGILE score was 3.00 (range: 0–10). Based on the Fr-AGILE scoring system, 12.9% (*n* = 13) of the patients were categorized as robust, 54.4% (*n* = 55) as mildly frail, 30.7% (*n* = 31) as moderately frail, and 2.0% (*n* = 2) as severely frail.

Demographic characteristics and results of the comprehensive geriatric assessment are summarized in Table 1.

**Table 1:** Demographic and characteristic features of the study population

	Normal (*n* = 13)	Mild frailty (*n* = 55)	Moderate frailty (*n* = 31)	Severe (*n* = 2)	All of them	*p*
**Age**		76.20 ± 6.77	79.83 ± 5.70	76.00 ± 2.82	77.12 ± 6.39	0.022
**Gender**,** female**,** n (%)**	5 (5)	30(29.7)	25(24.8)	2(2)	62(61.4)	0,018
**Education**,** n (%)** <5 years 5–8 years >8 years	6 (5,9) 5 (5) 2 (2)	36 (35,6) 5 (5) 14 (13,9)	26 (25,7) 0 (0) 5 (5)	2 (2) 0 (0) 0 (0)	70 (69,3) 10 (9,9) 21 (20,8)	**0**,**007**
**With whom he/she lives**,** n (%)** Alone With spouse With their children Other	1 (1) 8(7,9) 2 (2) 2 (2)	8 (7,9) 25 (24,8) 13(12,8) 9 (8,9)	6 (5,9) 6 (5,9) 17 (16,8) 2 (2)	0 (0) 2 (2) 0 (0) 0 (0)	15 (14,9) 41 (40,6) 32 (31,6) 13 (12,9)	0,106
**BMI**	24.8 (22–32,4)	24 (14–45)	23,4 (20–41)	24(22–26)	24,1 (14–45)	0,215
**Cigarettes**,** n (%)** Not used Ex-Smoker Active Smoker	10 (9,9) 3 (3) 0 (0)	29 (28,7) 19 (18,8) 6 (5,9)	28 (27,7) 1 (1) 2 (2)	2 (2) 0 (0) 0 (0)	69 (68.3) 23 (22.8) 3 (2.9)	0,060
**Number of diseases**	2 (0–4)	2 (0–5)	2 (0–8)	2 (0–3)	2 (0–8)	0,724
**Number of drugs**	4,61 ± 3,20	4,94 ± 2,95	5,00 ± 3,25	8,0 ± 5,65	5,20 ± 3,14	0,445
**Number of falls**	0 (0–2)	0 (0–3)	0 (0–10)	0,5 (0–1)	0,4 (0–10)	0,116
**Urinary Incontinence**,** n (%)**	8 (7,9)	21 (20,8)	11(10,9)	0 (0)	40 (39,6)	0,241
**Katz ADL score**	5 (5–6)	6 (4–6)	5 (1–6)	5 (5–6)	5 (1–6)	0,007
**Lawton-BrodyIADL score**	8 (5–8)	8 (1–8)	5 (0–8)	5 (5–5)	4 (0–8)	**< 0**,**001**
**MMSE score**	28 (25–30)	27 (13–30)	22 (9–29)	24 (23–26)	25 (9–30)	**< 0**,**001**
**MNA-SF score**	14 (6–15)	13 (7–14)	10 (3–14)	9,5 (5–14)	11 (3–14)	**< 0**,**001**
**Yesavage GDS score**	2,23 ± 2,21	2,90 ± 2,55	4,84 ± 4,18	6,50 + 4,95	3,48 ± 3,19	0,054
**Handgrip**,** average SD**	22,16 ± 9,58	17,98 ± 9,77	17,43 ± 9,82	15,05 + 9,82	18,28 ± 12,22	0,008

*ADL: Activities of Daily BMI: Body mass index*,* Living GDS: Geriatric Depression Scale*,* IADL: Instrumental Activities of Daily Living*,* MMSE: Mini-Mental State Examination MNA-sf: Mini Nutritional Assessment short-form*,,* SD: Standard deviation*

Inter-rater reliability for the items in the Fr-AGILE scale was evaluated and is summarized in Table 2. Kappa values between 0.61 and 0.80 were interpreted as indicating substantial agreement, while values between 0.81 and 1.00 indicated excellent agreement. Congruent internal consistency coefficients were observed across all domains. Specifically, substantial agreement was found in the physical, mental, and nutritional domains, and excellent agreement was observed in the socioeconomic domain.

**Table 2:** Cronbach’s alpha internal consistency coefficients of Fr-AGILE scale subheadings

Agile Scale subheadings	Internal consistency coefficients
**Physical**	0.773
**Mental**	0.799
**Nutrition**	0.791
**Socioeconomic**	0.823
**Total**	0.783

*calculated based on total scores.

Table 3 The fr-AGILE score was statistically correlated with FRAIL, Clinical Frailty Score. The fr-AGILE score showed significant correlations with specific frailty-related domains of the Comprehensive Geriatric Assessment, particularly functional status, activities of daily living, and comorbidity burden, rather than with the CGA as a whole.

**Table 3:** Correlation with other scales

	Scale correlation	*P*
**FRAIL Score**	0.742	< 0,001
**Clinical Frailty Score**	0.732	< 0,001
**Comprehensive Geriatric Assessment**	0.911	< 0,001

The Fr-AGILE scale assessed frailty across four domains: physical (e.g., “Do you feel that you have to exert effort for everything?“, “Do you need help going up and down stairs?“, and handgrip strength), mental (e.g., time disorientation, delayed recall, and “Do you feel depressed?“), nutritional (e.g., unintentional weight loss > 4.5 kg in the past year, need for assistance while eating), and socioeconomic (e.g., receiving financial or physical assistance from family members). It was statistically demonstrated that each of these domains significantly contributed to identifying vulnerability within their respective groups (Table 4).

**Table 4:** Fr-AGILE scale scores of all patients

	All (*n* = 101)	Not fragile (*n* = 13)	Slightly Fragile (*n* = 55)	Medium Fragile (*n* = 31)	Severe Fragile (*n* = 2)	Total correlation	When KR-20 is deleted	*p*
**1.Do you feel that you have to exert effort for everything? (%)**	34 (33.7)	0 (0)	12 (11.9)	20 (19.8)	2 (2)	0.440	0.645	< 0.001
**2.Do you feel that you need help going up and down the stairs (%)?**	50 (49.5)	0 (0)	19 (18.8)	29 (28.7)	2 (2)	0.538	0.611	< 0.001
**3.Hand Squeeze Strength (1) (%)**	39 (38.6)	0 (0)	16 (15.8)	22 (21.8)	1 (1)	0.226	0.680	< 0.001
**4.Time disorientation (2) (%)**	7 (6.9)	0 (0)	2 (2.0)	4 (4.0)	1 (1.0)	0.204	0.685	< 0.001
**5.Delay in recall (3) (%)**	55 (54.5)	0 (0)	28 (27.7)	25 (24.8)	2 (2)	0.173	0.670	< 0.001
**6. Do you feel depressed (%)?**	22 (21.8)	0 (0)	7 (6.9)	13 (12.9)	2 (2)	0.246	0.654	< 0.001
**7. Have you experienced weight loss of more than 4.5 kg in the last year (%)?**	28 (27.7)	0 (0)	13 (12.9)	13 (12.9)	2 (2)	0.125	0.701	< 0.001
**8. Do you get help while eating? (%)**	7 (6.9)	0 (0)	0 (0)	5 (5)	2 (2)	0.182	0.669	< 0.001
**9. Do you receive financial assistance from family members (%)?**	11 (10.9)	0 (0)	3 (3.0)	6 (5.9)	2 (2.0)	0.151	0.678	< 0.001
**10.Do you receive physical help from family members? (%)**	36 (35.9)	0 (0)	9 (8.9)	26 (25.7)	1 (1)	0.480	0.625	< 0.001

**Fig. 1:**
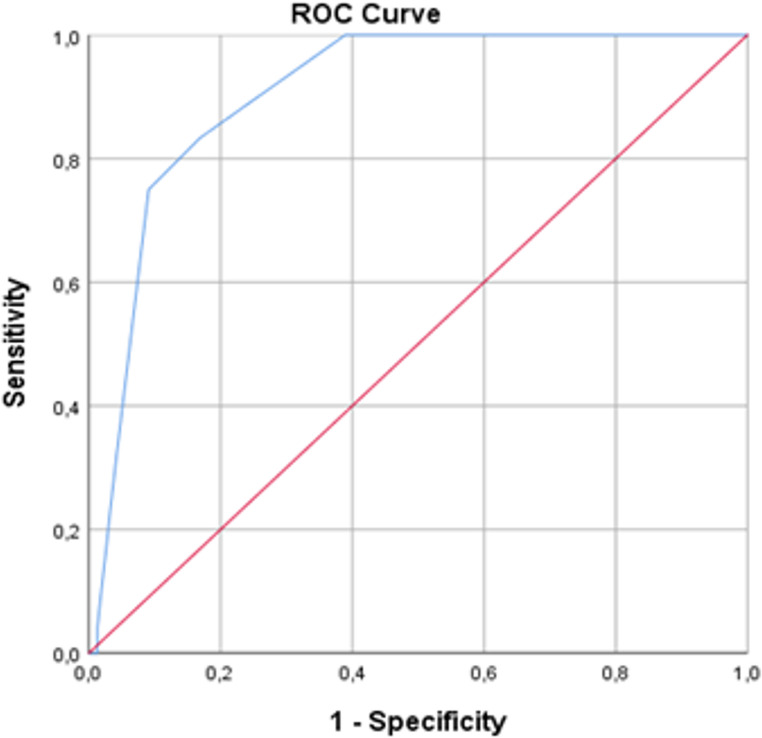
ROC analysis of the Fr-AGILE Scale *AUC: 0.906, p<0.001AUC, Area under the curve; ROC, receiver operating characteristic curve*

The ROC curve illustrating the predictive ability of the Fr-AGILE scale for identifying frailty is presented in Fig.1 The area under the curve (AUC) was calculated as **0.906**, indicating excellent diagnostic accuracy (*p* < 0.001). Using the Clinical Frailty Scale as a reference, the **sensitivity** and **specificity** of the Fr-AGILE scale were found to be **71.4%** and **94.1%**, respectively. The **positive predictive value (PPV)** was **83.3%**, while the **negative predictive value (NPV)** was **56.1%**.

## Discussion

This study statistically confirmed the validity and reliability of the Fr-AGILE frailty scale for use in the Turkish geriatric population. The findings demonstrate that Fr-AGILE is an effective tool for identifying frailty across multiple domains—physical, mental, nutritional, and socioeconomic—within a short time frame of approximately 3 to 5 min. It’s rapid and easy administration makes it particularly suitable for routine use in outpatient clinical settings to assess frailty in older adults. Our findings suggest that the Fr-AGILE scale is an effective predictor of fraility risk, especially mental, physical, socioeconomic and nutritional areas in geriatric outpatients. In the rapid assessment of geriatric patients thought to be at risk of frailty, the Fr-AGILE scale can be used in outpatient, emergency department, or inpatient visits because it provides a rapid and effective risk profile.

The findings of our study demonstrated high internal consistency of the Fr-AGILE scale, confirming its internal reliability. Inter-rater reliability between two independent assessors was also established. Congruent internal consistency coefficients were observed across all domains coefficients were observed across all frailty domains, with substantial agreement in the physical, mental, and nutritional domains, and excellent agreement in the socioeconomic domain, indicating robust reliability across different evaluators.

There are several established tools for assessing frailty. While the **Fried Frailty Phenotype** is widely accepted as a gold standard, its comprehensive questionnaire may lead to low patient compliance and prolong the assessment process. Similarly, although the **Comprehensive Geriatric Assessment (CGA)** is effective in identifying frailty, it requires significant time and clinician effort to complete. These limitations have highlighted the need for more practical tools, particularly in outpatient settings.

The **FRAIL Scale**, which served as a reference measure in our study, has previously been validated in the Turkish population. It has demonstrated significant clinical correlation with the CGA in earlier research [23, 24]. In our study, results obtained from the FRAIL Scale were found to be consistent with those of the Fr-AGILE scale.

A key strength of the Fr-AGILE scale is its ability to assess additional domains—**socioeconomic** and **cognitive (mental)**—which are not addressed by the FRAIL Scale [24]. Furthermore, the Fr-AGILE’s validity and reliability results were also significantly correlated with the **Clinical Frailty Scale (CFS)**. Unlike the CFS, which relies largely on the physician’s subjective judgment, the Fr-AGILE provides a more structured and objective evaluation through standardized patient-directed questions. This multidimensional approach—covering physical, mental, nutritional, and socioeconomic domains—makes the Fr-AGILE a valuable and practical tool for routine clinical use. In the rapid assessment of geriatric patients, the Fr-AGILE scale can be used in outpatient, emergency department, or inpatient visits.

The adaptation of an existing scale offers several advantages. Utilizing a tool that has already been applied and validated in another country facilitates knowledge transfer and enables future **cross-cultural comparisons** in geriatric research [25].

According to a review by Jorgensen et al. on frailty screening in patients aged 65 years and older admitted to the emergency department [26], frailty was assessed across four domains. The findings revealed that patients identified as frail at initial admission had significantly longer emergency department stays, higher hospitalization rates, and increased mortality. These results underscore the clinical value of a frailty assessment tool that is both **multidimensional** and **time-efficient**, making it suitable for use in fast-paced environments such as emergency departments.

While biological age is not a direct indicator of frailty, several studies have reported a correlation between advancing age and increased frailty. For example, in a study by Morley et al., which included individuals aged 49–65 years and assessed frailty using the FRAIL scale, activities of daily living, and gait speed, frailty was shown to increase with age [6]. Similarly, in our study, frailty levels tended to rise with increasing age up to the moderate frailty group. However, the mean age in the severe frailty group was comparable to that of the mildly frail group. This finding may be attributed to the study population being limited to patients attending outpatient clinics, whereas individuals with severe frailty are more likely to be followed in emergency or inpatient settings.

In contrast, a validity and reliability study conducted by Jung et al. in a Korean elderly population (mean age: 76.8 years) found no significant correlation between age and frailty [27]. These discrepancies highlight the potential influence of clinical setting and patient selection on the relationship between age and frailty.

The BMI scores of patients with and without frailty appeared to be similar; however, malnutrition risk was particularly observed among those with moderate to severe frailty. Similarly, in the study by Soysal et al., which investigated malnutrition in older obese patients, BMI scores were comparable between patients at risk of malnutrition and those without risk [28]. In the same study, malnutrition and malnutrition risk were more frequently observed in patients who were taking six or more medications, had less than five years of education, were widowed, had lower IADL scores, experienced more frequent falls, had higher scores on the Geriatric Depression Scale, and were female. These findings are consistent with the results of our study. This suggests that malnutrition may be associated with socioeconomic status and lifestyle factors. The increase in the number of falls and the lower handgrip strength observed may be linked to a predisposition to frailty. Through the Fr-AGILE test, key components of frailty such as falls, malnutrition, and depression can be rapidly identified, allowing timely intervention to prevent these conditions.

When the findings of our study are compared with the anthropometric data reported by Ates Bulut et al. in the Turkish elderly population, a notable consistency emerges [29]. In both studies, older women demonstrated lower handgrip strength, whereas men exhibited higher skeletal muscle mass and SMI values. However, in our study, BMI did not significantly differ across frailty groups, while Ates Bulut et al. reported higher body weight and fat mass particularly in older women. This discrepancy suggests that BMI alone may be insufficient to reflect frailty status, and emphasizes the importance of muscle mass and muscle strength—particularly handgrip strength—as more reliable indicators of vulnerability. Furthermore, both studies consistently demonstrated a decline in physical performance parameters (e.g., walking speed, handgrip strength) with advancing age, underscoring the central role of muscle loss and functional decline in the pathophysiology of frailty.

In a study by Xu et al., which investigated various risk factors for frailty using comprehensive geriatric assessment and laboratory parameters, no statistically significant association was found between gender and frailty, while a significant correlation was observed between advanced age and frailty [30]. Similarly, our study did not reveal a statistically significant difference in frailty prevalence between male and female participants.

However, several studies worldwide have reported a higher prevalence of frailty among women. These discrepancies May be attributed to differences in demographic characteristics and sampling methods. In our study, 61% of participants were female, which may have influenced the gender-related findings. Future studies with more balanced gender distribution may provide clearer insights into the relationship between gender and frailty.

Validation studies of the FRAIL scale conducted in Mexican and Korean populations have demonstrated that frailty tends to decrease with increasing education level [27, 31]. Consistent with these findings, our study showed that frailty was more prevalent among individuals with less than five years of education. Notably, both patients categorized as severely frail in our cohort had less than five years of formal education.

Frailty is also known to be highly prevalent among older adults residing in nursing homes. In a meta-analysis by Kojima et al., In the rapid assessment of geriatric patients thought to be at risk of frailty, the Fr-AGILE scale can be used in outpatient, emergency department, or inpatient visits because it provides a rapid and effective risk profile [32].

Which included five studies and a total of 3,528 participants, frailty was identified as a significant factor contributing to institutionalization [33]. However, since our study was conducted among outpatients, the number of nursing home residents was low, and no statistically significant association was found between living in a nursing home and frailty severity.

Interestingly, the two individuals categorized as severely frail were living with their spouses. This observation suggests that social support and living arrangements may not always mitigate frailty, and further studies with larger sample sizes are warranted to explore the role of caregiver dynamics and informal care in the progression of frailty.

Older adults are at increased risk of frailty due to reduced mobility and diminished engagement in both physical and cognitive activities. This association has also been supported by Baş et al., who reported a significant relationship between decreased physical activity and increased frailty levels [34]. However, in our study, no statistically significant difference was found between physical activity levels and frailty groups, likely due to the limited number of physically active participants.

Frailty and malnutrition frequently coexist in older populations [35]. Boulos, Salameh, and Barberger-Gateau demonstrated a direct relationship between frailty and undernutrition [36]. While our study investigated this association through weight loss, no significant difference was found across frailty groups. Notably, a higher proportion of participants in the moderate and severe frailty groups responded “don’t know” when asked about weight loss, potentially affecting accuracy. Nevertheless, when malnutrition risk was assessed using the Mini Nutritional Assessment (MNA), it was found to increase progressively with the severity of frailty. These findings highlight the Fr-AGILE scale’s utility in simultaneously identifying both frailty and nutritional vulnerability.

In addition, a study by Lee et al., which assessed frailty using the Fried criteria in 383 individuals aged 75 and older in a primary care setting, found a significant inverse relationship between handgrip strength and frailty [37]. In our study, a similar trend of decreasing handgrip strength with increasing frailty was observed, although the differences were not statistically significant. The limited sample size may have contributed to this lack of statistical power.

As expected, our study found that the **Lawton-Brody Instrumental Activities of Daily Living (IADL) score** declined progressively with increasing frailty severity. This aligns with the findings of Silva et al., who reported greater difficulty in performing routine daily tasks among frail older adults [38]. Similarly, a large-scale study involving 1,645 participants demonstrated that frailty is significantly associated with impaired functional capacity, leading to increased dependency in daily living activities [39]. These results reinforce the strong link between frailty and reduced functional independence in the geriatric population.

Mini-Mental State Examination (MMSE) scores, a key component of comprehensive geriatric assessment, were found to be lower among patients identified as frail by the Fr-AGILE scale in our study. This finding is consistent with a cross-sectional study conducted by Macuco et al. in Brazil, which involved 384 older adults and demonstrated that cognitive function declined as frailty severity increased These results support the inclusion of cognitive domains in frailty assessment tools and highlight the importance of multidimensional approaches like Fr-AGILE [40].

This study has several limitations. First, it was conducted on a relatively small sample of patients attending a single-center geriatrics outpatient clinic, which may limit the generalizability of the findings to the broader older adult population. A limitation of the test in terms of applicable population is that it cannot be used in patients who are unable to perform handgrip strength tests or who are unable to walk.

Despite these limitations, the study also has notable strengths. It was designed prospectively, allowing for real-time data collection and minimizing recall bias. Moreover, the Fr-AGILE scale was evaluated in accordance with established procedures for scale validation, including assessments of inter-rater reliability, test–retest reliability, and internal consistency. The multidimensional and time-efficient nature of the tool reinforces its clinical utility in routine outpatient practice.

## Conclusion

The Fr-AGILE scale demonstrated high inter-rater reliability and strong internal consistency across all domains. Substantial agreement was observed in the physical, mental, and nutritional domains, with excellent agreement in the socioeconomic domain. Based on these findings, the Fr-AGILE scale is a rapid, easy-to-administer, valid, and reliable tool for assessing frailty in older adults within the Turkish population. Its multidimensional structure makes it particularly suitable for routine use in outpatient geriatric practice.

## Data Availability

The data supporting the findings of this study are available from the corresponding author upon reasonable request.
